# Commercially Available Molecular Approaches to Evaluate Endometrial Receptivity: A Systematic Review and Critical Analysis of the Literature

**DOI:** 10.3390/diagnostics12112611

**Published:** 2022-10-27

**Authors:** Evangelos Maziotis, Theodoros Kalampokas, Polina Giannelou, Sokratis Grigoriadis, Anna Rapani, Marios Anifantakis, Amalia Kotsifaki, Agni Pantou, Olga Triantafyllidou, Despoina Tzanakaki, Spyridoula Neofytou, Paraskevi Vogiatzi, Panagiotis Bakas, Mara Simopoulou, Nikolaos Vlahos

**Affiliations:** 1 Department of Physiology, Medical School, National and Kapodistrian University of Athens, 75, Mikras Asias Str., 11527 Athens, Greece; 2 Second Department of Obstetrics and Gynecology, Aretaieion Hospital, Medical School, National and Kapodistrian University of Athens, 76, Vasilisis Sofias Avenue, 11528 Athens, Greece; 3 Centre for Human Reproduction, Genesis Athens Clinic, 14-16, Papanikoli Str., 15232 Athens, Greece; 4 Andromed Health & Reproduction, Fertility Diagnostics Center, 3, Mesogion Str., 15126 Athens, Greece

**Keywords:** endometrial receptivity, IVF, embryo transfer, implantation, implantation window, endometrium, biomarkers

## Abstract

Despite the advances in the field of reproductive medicine, implantation failure represents a challenging condition affecting 10–30% of patients subjected to in vitro fertilization (IVF). Research has focused on the identification of molecules playing crucial roles in endometrial receptivity, with the aim of designing predictive tools for efficient detection of the implantation window. To that end, novel molecular genomic and transcriptomic approaches have been introduced as promising tools to enable personalized approaches with the aim of optimizing embryo transfer dating. However, the clinical value of these approaches remains unclear. The aim of this study is to provide a systematic review and critical analysis of the existing evidence regarding the employment of commercially available novel approaches to evaluate endometrial receptivity. An Embase and PubMed/Medline search was performed on 1 February 2022. From the 475 articles yielded, only 27 were included and analyzed. The considerable heterogeneity of the included articles indicates the uniqueness of the implantation window, showcasing that the optimal time for embryo transfer varies significantly between women. Moreover, this study provides information regarding the technical aspects of these advanced molecular tools, as well as an analysis of novel possible biomarkers for endometrial receptivity, providing a basis for future research in the field.

## 1. Introduction

In an effort to enhance clinical outcomes of in vitro fertilization (IVF), numerous studies in the literature have investigated optimal embryo culture conditions [1,2], morphological criteria for embryo assessment [3,4], and the preferred day for embryo transfer [5,6,7]. These studies portray the astonishing progress in the field of assisted reproduction technology (ART) within the last 40 years, although live birth rates remain poor [8]. The phenomenon of embryo implantation is considered to be the “black box” of human reproduction, as it is a highly complex, multifactorial, and largely unknown procedure from a molecular standpoint. On one hand, the viability and quality of the embryo is undoubtably of paramount significance for successful implantation. On the other hand, the role of the endometrium is equally valuable in establishing the molecular dialogue required to achieve implantation. Several studies have suggested that impaired receptivity of the endometrium is a crucial factor resulting in implantation failure [9]. Endometrial receptivity (ER) is thought to contribute to two-thirds of implantation failure cases [10].

It is well-established that successful implantation requires a competent blastocyst, a receptive uterus, and a productive interaction between the two [11]. The endometrium may be described as a rather refractory status, with the exception of during the limited time frame during which the highest endometrial receptivity is observed—called the “implantation window” or “window of implantation” (WOI) [10]. This mid-secretory phase occurs 6–10 days following the luteinizing hormone (LH) peak is regulated by several cytokines, growth factors, and other key molecules for endometrial and embryonic development [12,13]. In artificial cycles employing hormonal replacement protocols, the window of implantation is expected to occur 4 to 7 days following progesterone (P) administration. During this period, the synchronized development of a competent embryo, along with the receptive endometrium, orchestrate numerous molecular pathways fundamental for a successful pregnancy, as well for regulating immunomodulatory factors and the immune protection environment [12]. Embryo implantation may be viewed as one of the most crucial steps of reproduction [14]. Implantation is the final step of a complex cellular and molecular “dialogue” that is established between the embryo and the endometrium. It has been reported that two key components are required for a successful implantation to occur, namely a viable embryo of good quality with a significant developmental potential and a uterus characterized by a receptive endometrium [15]. Successful implantation requires synchronous coordination and communication between embryonic development and endometrial receptivity status. Numerous morphological alterations can be observed in the endometrium during the implantation phase, as well as during pregnancy, that facilitate the embryo’s attempts to adhere to the endometrial wall and subsequently penetrate the epithelial layer. This phase of essential alterations and adjustments is called the “implantation window” or the “window of receptivity” (WOI) [16,17]. During the WOI, the endometrium constitutes a hormonally regulated tissue enabling embryo implantation. The endometrial tissue exhibits characteristics that facilitate embryonic implantation and therefore pregnancy initiation.

Implantation and pregnancy sustenance represent an ongoing field of research, as multiple pathologies are attributed to mechanisms instigating implantation impairment and poor pregnancy maintenance. This is especially true especially of RIF cases and, most importantly, idiopathic RIF, for which the cause failure of the “dialogue” between the embryo and the endometrium cannot be attributed to a probable cause. Recurrent implantation failure (RIF) constitutes one of the most challenging pathologies encountered in the field of human reproduction, raising concerns among researchers and clinicians alike. There is a lack of consensus regarding the exact definition of RIF; nonetheless, the most widely accepted definition refers to RIF as the failure to achieve a clinical pregnancy following at least three or more consecutive transfers of good-quality embryos in fresh or frozen cycles for woman younger than 40 years of age [18]. Hysteroscopy and the treatment of hydrosalpinxes have been proven to be effective tools for the management of RIF cases [19,20]. Whereas preimplantation genetic testing for aneuploidy (PGT-A) had been proposed as means to address RIF from the perspective of the embryo, most efforts are focused on how to better understand and improve the endometrium with intrauterine injection of platelet-rich plasma (PRP), endometrial injury, and granulocyte colony-stimulating factor (G-CSF), all of which have been proposed as promising strategies for endometrial receptivity improvement [21,22]. The RIF conundrum has made it clear that research should focus on recent technological advances to provide physicians with non-invasive tools to address and successfully manage RIF patients. Given the recent trend of genomics, with the employment of microarray technology and next-generation sequencing (NGS), along with advances in bioinformatics, researchers are finally equipped with potent tools that enable the simultaneous analysis of a plethora of up- and downregulated genes known to be involved in the phenomenon of endometrial maturation and receptivity. In the era of “omics”, numerous tools have been developed, and continuous research is being conducted to decipher the molecular identity of a receptive endometrium. Recent studies have illustrated the involvement of small non-coding RNAs (sncRNAs), particularly microRNAs (miRNAs), in the regulation of endometrium and the maturation stage [21,23,24]. Although these approaches are promising, there is a growing need for further research prior to their implementation in clinical practice. Furthermore, their role in enhancing the reproductive outcome in cases of RIF remains to be further explored and meticulously validated.

Over the years, the approach of understanding endometrial transcriptomics to indicate the appropriate implantation window has given rise to commercial tools that are promising for the promotion of implantation success and assisted reproduction outcomes. Current evidence may be viewed as sparse, which may explain the “red” light that the HFEA has applied to endometrial receptivity analysis [25]. Whereas adverse effects have not been reported to date and a number of retrospective studies have been published, the lack of high-quality evidence seems to prevent these molecular tools from being recommended for routine clinical practice. Despite the lack of evidence-based approval for clinical routine practice, these predictive tools are commonly offered as options for ART patients, giving rise to a debate among practitioners regarding application [26,27], with open questions remaining and conclusive results yet to be drawn from data. This ongoing debate served as the motivation to conduct the present study. The current systematic review provides a timely investigation of the available commercial tools employed to identify WOI. Herein, we report, for the first time, on the effectiveness of such tool in evaluating endometrial receptivity, determining the ‘implantation window’, and leading to improved clinical outcomes. An analysis on the strengths and weaknesses regarding the application of these advanced molecular tools is provided herein, as well as a systematic review with data extraction on the efficiency, clinical value, limitations, and overall appropriateness of the application of such services.

## 2. Materials and Methods

**Figure 1:**
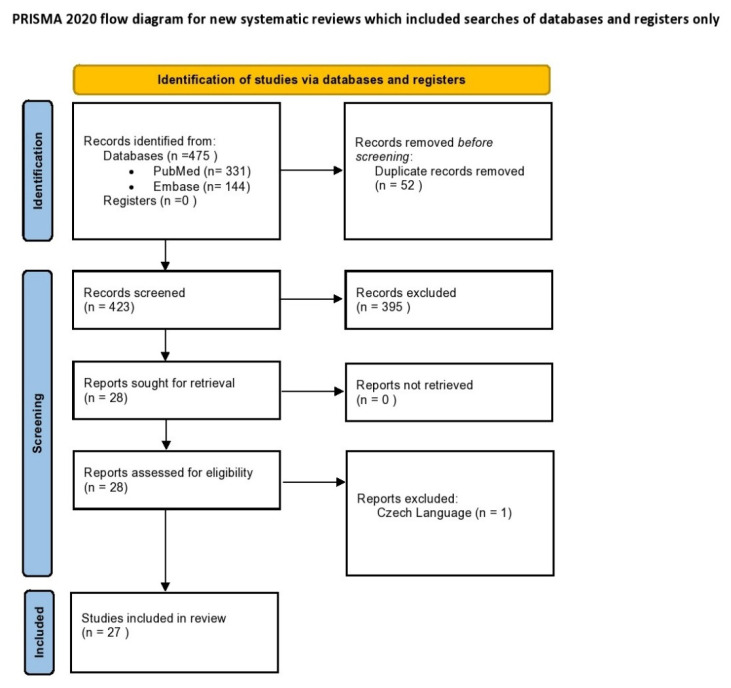
PRISMA flowchart of the study selection process.

A systematic search of the literature was performed in PubMed/Medline and Embase databases on 1 February 2022. The keywords employed and combined for the search strategy were: “In-Vitro Fertilization”, “IVF”, “Assisted Reproduction”, “Assisted Reproduction Techniques”, “Medical Assisted Reproduction”, “Intracytoplasmic Sperm Injection”, “ICSI”, “endometrial receptivity”, “window of implantation”, “window of receptivity”, “molecular”, “transcriptomic”, “genetic”, “gene expression”. The original search yielded 475 studies from the two databases. Following the removal of duplicate studies (*n* = 52), all records were screened, and full text was sought and obtained for relevant articles. Only studies that referred to commercially available tests for endometrial receptivity were included as part of the systematic review. The primary outcome measure was live birth rates. Secondary outcome measures were clinical pregnancy rate, accuracy, sensitivity, and specificity. Relevant articles were identified following title, abstract, and full-text screening, employing the flow chart of preferred reporting items for systematic reviews and meta-analysis (PRISMA) presented in Figure 1. The search was limited to full-length manuscripts published in English in peer-reviewed journals. One study was excluded, despite being relevant according to title and abstract screening, as the full-text manuscript was written in Czech. Screening and selection of literature was performed independently by three authors. Disagreements between the authors were resolved by an arbitration mediated by the senior authors. Citation mining was performed by employing the reference lists of all included articles and relevant reviews to identify other articles of relevance. A total of 27 studies were included in the present systematic review.

## 3. Results

### 3.1. Evolution of Commercially Available Tools for Endometrial Receptivity

Recurrent implantation failure constitutes a significant physiological “barrier” that hinders a successful outcome in IVF cycles, as women with high-quality embryos face this challenging condition following in vitro fertilization–embryo transplantation (IVF-ET) treatment. Therefore, receptivity deficiency seems to be a crucial factor entailed in this complex, pathophysiological phenomenon. Owing to the complex and unclear pathophysiology of RIF, a predictive and accurate tool is essential to estimate the window of implantation and ensure a successful embryo implantation.

Recently published data highlight a plethora of signaling pathways and their related genes that play key roles in the regulation of endometrial receptivity. In the era of “omics”, the evolution of microarray technology provides a powerful tool in the field of genetics, as well as in ART. Microarray technology combined with sophisticated bioinformatic data analysis allows for in-depth study of gene expression profiles, revolutionizing the concept of patient-tailored diagnosis and treatment [28]. This powerful predictive tool has been applied to numerous diseases, especially tumor classification [29,30], and in the past few years, has been introduced in ART in order to shed light on perplexing functions, such as ER [31,32].

The transcriptomic signature of human ER has been Investigated since 2002 [33], and numerous studies have been published reporting gene expression analysis in natural or controlled ovarian stimulation (COS) cycles for fertile and infertile women [34]. In the last decade, endometrial transcriptomics have been employed to enrich our knowledge of endometrial pathophysiology and to improve understanding of the implantation process. A considerable number of genes have been reported to be differentially expressed between the pre-receptive and the receptive endometrial phase. However, differentially expressed genes vary from study to study, ranging between 107 and 2878 genes [33,34,35,36,37,38]. A plausible explanation for this heterogeneity is the variety of patients recruited in the various studies. A review summarizing evidence regarding endometrial expression originating from the same patient in different endometrial phases reported that only two genes, namely osteopontin (SPP1) and interleukin 15 (IL-15), were differentially expressed [34]. A recent study reviewed numerous possible mucosal biomarkers, highlighting the importance of urocortin, activin A, interleukin 1beta (IL-1β), tumor necrosis factor alpha (TNF-α), interferon gamma-induced protein 10 (IP-10), and monocyte chemoattractant protein 1 (MCP-1), as well as oxidative stress biomarkers [39]. Furthermore, numerous genetic variations, as well as differential expression levels of genes or of non-coding RNAs, have been investigated as biomarkers of endometrial receptivity.

In the aforementioned studies, transcriptomic technologies have been employed to identify biomarkers present in the human endometrium. The role of these molecules in ER establishment has prompted researchers to identify the genomic signature of human endometrial receptivity, which could enable the detection of the WOI. As a result, based on the identified transcriptomic signature of ER, seven predictive tools have been commercialized. The aim of these tools is to navigate the embryo transfer procedure towards a more personalized approach by enabling clinicians to accurately identify the precise time of the WOI in a timely manner to increase implantation rates. Seven predictive tools are commercially available; three have been published in peer-reviewed papers, and two originate from the same study, whereas the Bioarray BioER has not been published in a peer-reviewed study. With respect to the Yikon ERT (YK-ERT), it is unclear whether the commercialized tool is identical to the published rsERT [40].

### 3.2. Window of Implantation Test (WIN-TEST)

In 2009, Haouzi and colleagues identified an exclusive transcriptomic signature by revisiting the global gene expression profile of human endometrial biopsies as presented in the literature [32]. In this study, the authors employed qRT-PCR to validate the expression levels of five genes specifically modulated during the WOI from the transcriptomic signature of 1012 genes. These five genes were *laminin.3* (LAMB3), *microfibrillar-associated protein5* (MFAP5), *angiopoietin-like 1* (ANGPTL1), *prokineticin 1* (PROK1), and *nuclear localized factor 2* (NLF2). When the expression of these five genes in the mid-secretory phase (LH + 7) was compared to the early secretory phase (LH + 2) in natural cycles, results indicated that all five endometrial genes were overexpressed [32]. Exploration of their roles in the pathophysiology of the uterus revealed that all five genes are involved in the extracellular-matrix remodeling of the endothelial-cell microenvironment, angiogenesis, and the formation of the endothelial fenestra [32]. The authors identified eleven genes, *MFAP5*, *ANGPTL1*, *PROK1*, *NLF2*, *LAMB3*, *BCL2L10*, *CD68*, *TRPC4*, *SORCS1*, *FST*, and *KRT80* that they proposed to be upregulated and predictive of a receptive endometrial status [41]. Therefore, a novel method for assessing endometrial receptivity was introduced; this predictive tool, the Win-Test (window implantation test) was the first of its kind to be commercialized [41].

To perform the Win-Test patients, are subjected to endometrial biopsy between the fifth and the ninth day following progesterone administration (P + 5 to P + 9) or between the sixth and the ninth day following luteinizing hormone surge (LH + 6 to LH + 9). The endometrial biopsy is rinsed in phosphate-buffered saline (PBS), placed in a cryotube containing RLT RNA extraction buffer, and frozen at −80 °C (dry ice or liquid nitrogen) until shipment on dry ice. The Win-Test results are provided within 5 days post reception of biopsies [32,38,41].

For an endometrium to be characterized as receptive, each gene among the eleven genes screened in the Win-Test is expected to be overexpressed compared to a pre-receptive endometrium [42]. In their most recent prospective study, Haouzi et al. reported that an endometrium is defined as receptive when the mean expression of the 11 genes is 70% or more of the expression levels of the positive control, partially receptive when the mean expression of the 11 genes is between 50 and 70%, and “non-receptive” when the expression levels are less than 50% of the positive control [42]. Consequently, the Win-Test predictive tool is based on the quantitative expression of the aforementioned 11 genes of the ER profile, coupled with an algorithm used to identify the receptive state. Further studies showcased that the frequency of non-receptive patients in the RIF population varies within the range of 25–88.5% [42,43,44].

Regarding clinical outcomes, two retrospective studies and one prospective study have been conducted. In one retrospective study, the authors identified three endometrial profiles indicative of observed receptivity changes in a 48 h interval (P + 6 and P + 8 days) [45]. The mean clinical pregnancy rate observed for the three profiles following pET was 21.82% (12 out of 55) per FET. The clinical pregnancy rate appears to significantly differ between the profiles (4/5 in type II and 0/13 in type III); however, the small sample size prevented the authors from performing statistical analysis. Another retrospective study employed the Win-Test in 15 RIF patients. The positive hCG rate was 60% (9/15), whereas the live birth rate was 33.3% (5/15). The authors proceeded with analysis of small non-coding RNAs (sncRNAs), comparing endometrial samples from women who gave live birth to women with negative hCG, reporting that a total of 261 sncRNAs were differentially expressed [43]. A prospective evaluation prospectively of the Win-Test in RIF patients, revealed that positive hCG, clinical pregnancy, ongoing pregnancy, and live birth rates were increased in associated with personalized embryo transfer (pET) compared to patients undergoing standard ET [42]. Although data seem promising, it should be emphasized that all of the abovementioned studies were conducted by the same research group; thus, future prospective studies from independent centers should be conducted to validate the efficacy of the Win-Test.

### 3.3. Endometrial Receptivity Array (ERA)

In 2011, Díaz-Gimeno and colleagues presented the endometrial receptivity array (ERA) to the scientific community, a molecular predictive tool implementing microarray technology to evaluate endometrial receptivity [31]. Development of the ERA test included identification of 238 genes that were differentially expressed in the transition from the pre-receptive status to the receptive status of the endometrium, along with the employment of artificial intelligence to assist in chronologically determining the implantation window of a given patient, regardless of histological examination [31]. This tool was designed to identify ER by comparing the genetic profile of a sample obtained 7 days following the LH serum peak (LH + 7) in a natural cycle or 5 days following progesterone administration (P + 5) in a hormone replacement cycle. Its predictive power is significant, with specificity and sensitivity levels of 0.8857 and 0.99758, respectively [31]. Additional studies reported lower predictive power than initially documented values, with an accuracy of 0.88, sensitivity of 0.90, and specificity of 0.97 [46]. The same research group evaluated the accuracy of the ERA test against the traditional histologic method and concluded that ERA is more accurate than endometrial histologic dating, considering the day of the LH peak as the reference point [47]. ERA as a predictive tool has been modified several times over the years by the manufacturer and recently became available as the NGS ERA Predictor 2.0 [46].

Following biopsy, the endometrial tissue is transferred to a cryotube containing RNA preservation buffer and stored at 4 °C or on ice for at least 4 h. Then, samples are shipped at room temperature for transcriptomic analysis, and the ER status is diagnosed by the ERA computational predictor [47,48]. The results are typically available within 2–3 weeks and indicate a receptive, an early receptive, a late receptive, a pre-receptive, a post-receptive, or a proliferative endometrium.

The ERA test constitutes the most investigated method for evaluation of the implantation window. However, most data regarding the ERA test are presented in retrospective studies, a number of which fail to include a control group. It should be highlighted that this is the sole endometrial receptivity test that has been assessed in an RCT to date. According to the results of the RCT, ERA did not significantly increase the probability of either clinical pregnancy or live birth compared to either fresh or frozen embryo transfers when an ITT analysis was performed [49]. Nonetheless, ERA seems to enhance cumulative pregnancy rates in a 12-month period. Moreover, when analyzed per ET, it seems that ERA significantly increases clinical pregnancy rates when compared to frozen ETs and is on the verge of statistical significance when compared to fresh ETs [49]. The small sample size of each group may be a reason for caution when interpreting the results of the RCT. Further RCTs, along with data pooling in a future meta-analysis, may reveal different results with respect to the clinical effectiveness of the ERA test. On the other hand, ERA may be more effective when implemented solely to patients with RIF, as evidenced by multiple retrospective studies, although this conclusion is yet to be confirmed by a prospective study or an RCT.

A total of 16 studies evaluating ERA were identified. Among these studies, 13 were retrospective in nature, whereas 2 were prospective, with 1 RCT. Among the 13 retrospective studies, only 4 employed a control group undergoing the standard ET protocol. The results collected in the retrospective studies report that ERA may be beneficial for both RIF patients and the general population. However, evaluation prospective studies and RCTs reveals that ERA may be beneficial mainly for RIF patients, as clinical pregnancy and live birth rates do no exhibit statistically significant results in the general population when employing ITT analysis. A summary of the available data for the ERA test is presented in Table 1.

### 3.4. ER Map/ER Peak

Given the encouraging ERA results, in 2016, a novel molecular tool was developed for the evaluation of endometrial receptivity. The ER Map employed RT-PCR for a panel of 16 genes implicated in endometrial proliferation and immune response. The initial results reported 100% accurate classification in 130 endometrial samples [64]. Another study from the same group employing 48 genes similarly reported 100% accurate classification from 260 endometrial samples [65]. As the aforementioned studies were presented as conference abstracts, a subsequent full-text published study was employed as the basis for both ER Map and ER Peak development, highlighting that 40 differentially expressed genes may provide accurate classification [66]. An endometrial biopsy is performed either on day LH + 7 in a natural cycle or on day P + 5 in a hormone replacement cycle. The shipment method is similar to that of ERA. In brief, following the biopsy, the endometrial tissue is transferred to a cryotube containing RNA preservation buffer and stored at 4 °C or on ice for at least 4 h. Then, the samples are shipped at room temperature for transcriptomic analysis.

In terms of clinical outcomes, each test has been validated in only one study. The ER Map was evaluated in a non-RIF population and resulted in 302 clinical pregnancies out of 681 patients employing pET (44.35% pregnancy rate), which is significantly higher compared to 15 out of 65 clinical pregnancies when the ET was performed outside the WOI. However, the ER Map has not been evaluated in comparison to the standard ET protocol [67]. The ER Peak was evaluated in a retrospective study analyzing the clinical pregnancy and live birth rates of 550 RIF patients. The study reported that 92 out of 244 patients undergoing pET presented with a clinical pregnancy compared to 59 out of 306 patients undergoing a standard ET protocol. Similarly, live birth rates were significantly higher following pET (29.9% vs. 9.5%). Despite the promising results, both tests lack robustness considering the lack of RCTs and prospective studies to validate their effectiveness.

### 3.5. BeREady Test

In 2019 Saare et al. employed a cost-effective method of targeted allele counting by sequencing (TAC-seq) to reduce the high cost of global expression profiling [68]. The basis for the development of this test was established two years earlier in a meta-analysis of possible endometrial receptivity biomarkers [23]. The authors explored the role of a panel of 57 ER genes as potential biomarkers according to the results of the aforementioned meta-analysis in order to determine the exact molecular menstrual cycle phases of endometrial samples [68]. As a result, the authors proposed the introduction of a novel predictive tool aiming to specify the precise menstrual cycle phase of endometrial samples from uncertain cycle phases that may benefit studies investigating true disease-related markers [68].

The commercialized predictive tool, named the, uses an innovative method of targeted allele counting by sequencing (TAC-seq) [69] to assess the expression of 67 biomarker genes that indicate a receptive endometrium. Similarly to other commercialized tests, the endometrial sample is characterized as pre-receptive, early-receptive, receptive, late-receptive, or post-receptive. A clinician performs an endometrial biopsy with a Pipelle in an affiliated clinic, and then the samples are dispatched for assessment and biomarker analysis. The results are available within 14 business days. However, no studies have been conducted to evaluate the efficiency of the BeREady test in accurately predicting the implantation window or its effect on clinical outcomes.

### 3.6. The Future of Transcriptomic Analysis for Identification of Endometrial Receptivity (ER)

During the past two years, a considerable number of studies have been published investigating the molecular and transcriptomic profile of the endometrium and focusing on changes during the WOI. Among the 331 studies that were retrieved from PubMed employing the keywords listed in the Materials and Methods section, 104 (31.42%) were published in 2020 or later. To put this into perspective, the first commercially available tools were developed more than a decade ago. Taking this into consideration, along with the fact that research in this field is ongoing, it can be extrapolated that the currently available tools may be subject to future calibrations, and novel predictive tools may be designed and marketed in the future. Although the present systematic review is focused strictly on commercially available tools, novel biomarkers may assist in shaping future predictive tools and are therefore briefly analyzed herein.

As embryo implantation is an immunologically based procedure, biomarkers can be indicative of endometrial receptivity. Specifically, CD56+ levels have been associated with endometrial receptivity [70]. Immunological profiling for endometrial receptivity has been observed to present with similar results to endometrial receptivity predictive tools. The simultaneous employment of both of these methods was recently reported to present with higher implantation rates per embryo transferred; however, no statistically significant difference was observed when evaluating clinical pregnancy per patient [63]. Owing to the small sample size and the fact that a trend was observed, further investigation is required to evaluate the effectiveness of the abovementioned combination. Nonetheless, this recent data may serve as a basis for the emergence of a new era of transcriptomic analysis with respect to endometrial receptivity.

Apart from the immunological changes during the receptive phase of the endometrium, studies employing global transcriptomic or proteomic analysis have reported that numerous proteins and mRNAs are differentially expressed depending on endometrial receptivity status. Employing uterine fluid (UF), it was observed that 2247 transcripts [71], 367 proteins [72], and 61 miRNAs targeting 15 pathways [73] were differentially expressed with respect to endometrial receptivity. One of the pathways that has been suggested as predictive for IVF outcome is the Toll-like receptor (TLR) signaling pathway. TRIB2 and TLR9 were differentially regulated in RIF patients compared to healthy controls, highlighting their potential employment as biomarkers for RIF [74]. The expression of TRIB2, TLR9, ICAM1, NFKBIA, VCAM1, LIF, VEGFB, and TLR5 was observed to be significantly altered between pre-receptive and receptive endometrial status, indicating their involvement in endometrial receptivity [74]. One of the genes of particular interest that is regulated by the TLR signaling pathway is LIF, as it has been associated with pinopode development [75]. The association of pinopodes with endometrial receptivity had been a matter of debate over the past decade; however, hitherto, their role in embryo implantation has not been clarified [76]. Another gene that has been associated with pinopode development is HOXA10. A study investigating the expression levels of LIF and HOXA10 in women with antiphospholipid antibodies and RIF concluded that both genes present with significantly decreased expression levels compared to healthy women during the WOI [75]. HOXA10 has been a gene of interest examining RIF patients, as it has been observed that its inhibition may contribute to the pathogenesis of RIF [77]. The gonadotropin-releasing hormone (GnRH) antagonist protocol has been observed to reduce both the expression levels and the protein levels of HOXA10, as well as S100P, two genes that have been associated with endometrial receptivity [78]. Further research is required to confirm the role of these genes in endometrial receptivity, as well as to evaluate the effect of different protocols on their expression levels.

As evidenced, small non-coding RNAs, especially miRNAs, are involved in the process of embryo implantation. However, their role in endometrial receptivity in women experiencing RIF remains unclear. The expression levels of miR-30d-5p in the endometrium were decreased during the implantation window, and it was observed that miR-30d-5p suppresses cytokine signaling 1 (SOCS1) and LIF expression, contributing to the pathophysiology of RIF [79]. Another miRNA, miR-185b, has been observed to be associated with implantation failure by disrupting HOXA10 expression. The circular non-coding RNA hsa_circ_001946 has been observed to impede the function of miR-185b and promote HOXA10 expression, contributing to a more receptive endometrium [80].

Whereas a vast numbers of genes and miRNAs have been observed to be differentially expressed, their exact role in the physiology of endometrial receptivity has not yet been delineated. Recent data indicate that in the midst of the overwhelming population of candidate genes that have been suggested to be implicated in the numerous pathways involved in ER, some specific genes are commonly acknowledged to play a more important role than others, such as LIF and HOXA10. Further investigation is required to elucidate the role of the identified genes and biomarkers in the physiology of ER, as they could represent potential targets for the enhancement of infertility treatments, especially in RIF women.

## 4. Discussion

Over the past 20 years, scientific research has been focused on deciphering the “black box” of the human implantation process. Studies employing transcriptomic analysis and molecular investigation of the endometrium have shed light and led to the emergence of predictive tools for clinicians in an effort to achieve improved clinical outcomes. A plethora of published studies showcase that the WOI is coupled with a specific transcriptomic profile. Four tools under five brand names have been developed, peer-reviewed, and commercialized, namely the Win-Test, the ERA test, the ER Map/ER Peak, and the BeREady test. The aim of these commercially available tests is to assess multidimensional endometrial receptivity to detect the WOI and therefore indicate the optimal time to perform the embryo transfer procedure. An interesting point of discussion is that although these tools are marketed as “diagnostic”, they are better described as “predictive”. We suggest this critical distinction in definition because these tools are employed to predict the implantation window of the menstrual cycle following the menstrual cycle during which the biopsy is performed. This is a clear prediction rather than a diagnosis; hence, the scientific community, along with patients should, acknowledge these tools as such.

In addition, two novel tools have been proposed for endometrial receptivity prediction. In 2021, He and colleagues reported on the development of a predictive tool, rsERT, employing transcriptomic analysis via RNA sequencing [40]. Whereas the development of this tool has been supported by Yikon, it is unclear whether it is identical to YK-ERT or a different version that may have not been commercialized yet. The development and evaluation of this test was published in one two-phase study. The development was retrospective in nature, as anticipated, whereas the validation was prospective in nature. The data from the validation phase reported a significant increase in clinical pregnancy rates in RIF patients (27/56 employing pET vs. 25/86 employing a standard ET protocol). On the other hand, no statistically significant difference was observed when comparing live birth rates (24/56 employing pET vs. 23/86 employing standard ET protocol) [40]. However, the small sample size does not allow definitive conclusions to be drawn. Furthermore, another novel endometrial receptivity panel was recently introduced, although it is not yet commercially available. The transcriptomic endometrial dating (TED) model is based on the differential expression of 73 genes, and as reported, it can accurately classify the receptivity of the endometrium [81]. However, the TED model has not yet been clinically evaluated.

**Figure 2:**
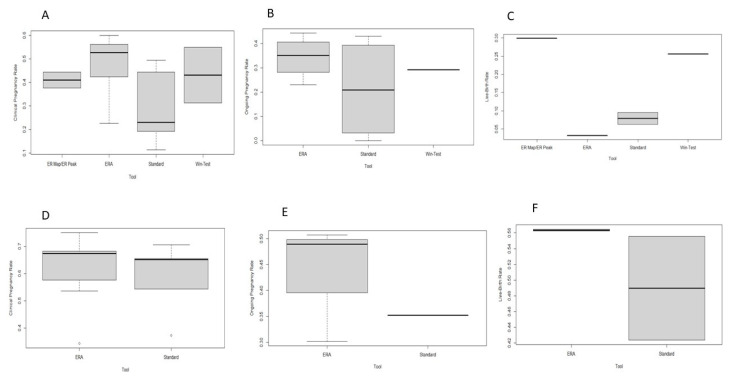
Boxplot of the clinical outcomes. (**A**): Clinical Pregnancy rates in RIF patients; (**B**): Ongoing Pregnancy rates in RIF patients; (**C**): Live-birth rates in RIF patients; (**D**): Clinical Pregnancy rates in non-RIF patients; (**E**): Ongoing Pregnancy rates in non-RIF patients; (**F**): Live-birth rates in non-RIF patients.

The timeframe of the WOI constituted added-value information in performing an embryo transfer; although all identified endometrial receptivity tests present with promising results, robust conclusive data and molecular calibration of evaluation methods originating from high-quality studies are required prior to conclusively declaring them as tools that enhance IVF outcomes. This fact justifies the red-light ranking by the HFEA traffic-light system and classification of this service as an add-on. The four prediction tests analyzed in the present study have the potential to assist clinicians in determining the exact time of the WOI via gene expression analysis; however, they exhibit distinct differences. According to our systematic review of the literature, the ERA test appears to be the most studied tool by the scientific community. Hitherto, only the ERA has been externally evaluated in an RCT, albeit a singular trial reporting limited evidence. The remaining tests have been evaluated mainly in retrospective studies and primarily by the groups that developed and introduced them for commercial use, resulting in inevitable bias. A box plot representing the clinical outcomes of the included studies is presented in Figure 2.

With respect to the genes included in the panels of the commercially available tests, the ERA explores the expression of 238 genes compared to the Win-Test, which analyzes 11; the ER Map/ER Peak, which analyzes 48; and the BeREady test, which analyzes 67 genes. The volume of information on gene expression provided by the ERA test may offer important insight into the transcriptomic profile of the patient. On the other hand, the BeREady test may represent a more cost-effective approach, as it incorporates a methodology of targeted allele counting by sequencing (TAC-seq) to reduce the high cost of assessing global expression profiling, indicating that cost-effectiveness studies are required in order to further explore such differences. As mentioned by Enciso et al., ER Map only shares seven common genes with the ERA test [66]; therefore, future studies should compare the accuracy of these tests in determining the implantation window, as well as the endometrial status.

A major limitation of the available endometrial receptivity tests is the lack of precise definition of the populations for which they are suitable. According to the published prospective studies, it remains uncertain whether these tools should be employed for patients undergoing frozen embryo transfers, whereas evidence suggest that they might be more beneficial for use in RIF patients. However, as previously mentioned, the RIF population is highly heterogenous, and numerous definitions have been employed for RIF. RIF is a pathology that may only be applied to IVF patients. The term RIF has been employed to describe patients with high-quality embryos that fail to implant. The discrepancies in definitions relate to the number of high-quality embryos, the number of ETs, and the definition of implantation. In several studies, implantation failure refers to a negative hCG test. However, according to other studies, implantation failure may refer to the absence of a gestational sac [82]. Furthermore, numerous risk factors for RIF have been identified, ranging from lifestyle factors, namely smoking, BMI, and alcohol consumption; and uterine factors, namely endometriosis, polyps, adenomyosis, and maternal age; to genetic factors, such as thrombophilia. A recent mathematical model includes the “overuse” of RIF definition, suggesting that RIF should not be employed if euploidy and maternal age are not accounted for [83]. It is important to note that the abovementioned studies do not employ a universal definition for RIF, and most do not account for euploidy or maternal age. This may represent a limitation when interpreting the findings of the present systematic review. It is imperative for the scientific community to reach a consensus regarding the definition of RIF and for future studies to determine the impact of ploidy status and age on RIF misclassification.

An additional cohort of patients that has been suggested to benefit from the implementation of these tests is women presenting with PCOS. According to studies employing transcriptomic analysis, the endometrial receptivity of women presenting with PCOS could be evaluated and assessed based on gene expression [84]. A recent study concluded that genes affecting glucose metabolism, compensatory hyperinsulinemia from an insulin-resistant state, and hyperandrogenism may affect endometrial receptivity in PCOS women [85]. Nonetheless, none of the currently commercially available endometrial receptivity tests has been evaluated in this population hitherto. Future studies should be performed in order to delineate whether receptivity evaluation is an angle worth exploring for the management of infertility issues faced by PCOS patients.

It is of paramount significance to design and conduct further external prospective studies, most importantly RCTs, including a control group of patients undergoing the standard ET protocol prior to recommending ER tests as beneficial for the general population or for specific population groups. We therefore encourage the design of future studies that could provide conclusive data. Owing to the nature of the reviewed predictive tests, blinding may not be employed in RCTs. In order to address this source of bias, a different type of study should also be performed, such as studies where all patients undergo endometrial biopsy; however, ET should be performed according to the standard protocol and not the evaluated predictive tool. Then, employing the known implantation data, the true-positive, true-negative, false-positive, and false-negative prediction of these tests should be calculated. Thus, the sensitivity, specificity, and positive and negative predictive value, as well as the area under the curve (AUC), can be determined. As these tests are provided as predictive tools, reporting on their true predictive values should be an absolute requirement to provide high-quality evidence. The resulting data of several studies designed as per the model proposed above should be summarized in a meta-analysis. Such an approach entailing meta-analytical evidence could safely lead to shaping future guidelines. When national authorities and international societies, such as HFEA, ESHRE, and ASRM, are in a position to rely on solid evidence, recommendations and guidelines to define evidence-based practice can be issued.

In addition to robust reporting on effectiveness, in order to soundly implement these tools in clinical practice to enhance clinical pregnancy rates on the grounds of proper research methodology, it is imperative to define the cohort and profile of patients that could benefit from their application. Women experiencing implantation failure in previous cycles or patients with RIF, all women undergoing ART treatments, women with unexplained infertility, or patients with underlying endometrial inflammatory-based defects, such as endometriosis and, in some cases, women with certain anatomically specific features, could constitute potential cohorts of patients for whom these commercial tools could revolutionize treatment and improve expected outcomes. Thus, even if data buttressing the efficiency of ER predictive tests is ascertained, perhaps the most important question raised appears to be how to identify which category of patients will benefit the most and which pathologies these services should be addressing. In the era of precision medicine, there is an urgent need for profiling in order to appropriately address the needs of every patient. This new study field of ER assessment needs to provide robust data before future horizontal application of these advanced molecular tools may claim to successfully enable individualization of fertility treatments and meet patients’ needs. Future well-designed prospective studies including control groups characterized by well-defined outcome measures of significant clinical impact, namely live birth rates, are imperative to conclude whether the introduction of endometrial receptivity evaluation into clinical routine practice could be safely justified.

To the best of our knowledge, this is the first contribution collectively reporting on commercially available tools for ER assessment. This systematic review enabled a comparison of clinical outcomes and technical aspects, and we also disused future directions for the evaluation of ER. The definition of the optimal population that may benefit from the application of such tests was also considered, highlighting the challenges associated with categorization and the importance of profiling. In this study, we present analysis of reported data for use by practitioners, highlighting the areas that demand further improvement with respect to the development and validation of predictive ER tools.

## Figures and Tables

**Table 1:** Summary of evidence regarding the efficiency of the ERA test.

Study	pET RIF	pET Without RIF	RIF (Control)	Without RIF (Control)	Outcome	Study Design
Ruiz-Alonso, 2013 [48]	9/16	6/8	N/A	N/A	Clinical pregnancy	Prospective multicenter
Ruiz-Alonso, 2014 [50]	123/205	N/A	12/52	N/A	Clinical pregnancy ^1^	Retrospective
	91/205	N/A	0/52	N/A	Ongoing pregnancy ^1^	
Mahajan, 2015 [51]	28/66	38/66	N/A	N/A	Clinical pregnancy	Retrospective
Hashimoto, 2017 [52]	25/44	N/A	N/A	N/A	Clinical pregnancy	Retrospective
Tan, 2018 [53]	N/A	36/71	N/A	N/A	Ongoing Pregnancy ^2^	Retrospective
Bassil, 2018 [54]	N/A	16/53	N/A	177/503	Ongoing pregnancy	Retrospective
Neves, 2019 [55]	N/A	14/24	N/A	84/119	Clinical pregnancy ^3^	Retrospective
	N/A	11/32	N/A	103/158	Clinical pregnancy ^4^	
Patel, 2019 [56]	116/220	N/A	N/A	N/A	Clinical pregnancy	Retrospective
Simon, 2020 [49]	N/A	58/80	N/A	50/92	Clinical pregnancy	RCT
	N/A	45/80	N/A	39/92	Live birth	
Cohen, 2020 [57]	21/93	N/A	N/A	N/A	Clinical pregnancy	Retrospective
	3/93	N/A	N/A	N/A	Live birth	
Cozzolino, 2020 [58]	72/195	N/A	1147/3227	N/A	Ongoing pregnancy ^5,7^	Retrospective
	9/27	N/A	110/255	N/A	Ongoing pregnancy ^6,7^	
Barrenetxa, 2021 [59]	N/A	58/85	N/A	N/A	Clinical pregnancy	Retrospective
Fodina, 2021 [60]	8/22	N/A	32/72	N/A	Clinical pregnancy ^5^	Retrospective
	40/72	N/A	43/87	N/A	Clinical pregnancy ^6^	
Eisman 2021 [61]	13/28	189/280	N/A	N/A	Clinical pregnancy	Retrospective
	6/26	137/280	N/A	N/A	Ongoing Pregnancy	
Riestenberg 2021 [62]	N/A	99/147	N/A	53/81	Clinical pregnancy	Prospective single-center
	N/A	83/147	N/A	45/81	Live birth	
Jia 2022 [63]	N/A	15/28	N/A	19/51	Clinical pregnancy	Retrospective

^1^ Data on first attempt only; ^2^ incomplete reporting of live birth; ^3^ autologous; ^4^ donors; ^5^ without PGT-A; ^6^ following PGT-A; ^7^ data are presented per ET cycle; RCT: randomized controlled trial.

## Data Availability

Not applicable.
